# Spiritual care at the end of life in the primary care setting: experiences from spiritual caregivers - a mixed methods study

**DOI:** 10.1186/s12904-019-0484-8

**Published:** 2019-11-09

**Authors:** Ian Koper, H. Roeline W. Pasman, Bart P. M. Schweitzer, Annemieke Kuin, Bregje D. Onwuteaka-Philipsen

**Affiliations:** 1 Department of Public and Occupational Health, Amsterdam UMC, Vrije Universiteit AmsterdamAmsterdam Public Health research institute, Amsterdam, The Netherlands; 2 Spiritual caregiver, Dijklander Hospital, Hoorn and Purmerend, The Netherlands

**Keywords:** Spiritual care, Primary care, Palliative care, Quality of care

## Abstract

**Background:**

Spiritual care is an important aspect of palliative care. In the Netherlands, general practitioners and district nurses play a leading role in palliative care in the primary care setting. When they are unable to provide adequate spiritual care to their patient, they can refer to spiritual caregivers. This study aimed to provide an overview of the practice of spiritual caregivers in the primary care setting, and to investigate, from their own perspective, the reasons why spiritual caregivers are infrequently involved in palliative care and what is needed to improve this.

**Method:**

Sequential mixed methods consisting of an online questionnaire with structured and open questions completed by 31 spiritual caregivers, followed by an online focus group with 9 spiritual caregivers, analysed through open coding.

**Results:**

Spiritual caregivers provide care for existential, relational and religious issues, and the emotions related to these issues. Aspects of spiritual care in practice include helping patients find meaning, acceptance or reconciliation, paying attention to the spiritual issues of relatives of the patient, and helping them all to say farewell. Besides spiritual issues, spiritual caregivers also discuss topics related to medical care with patients and relatives, such as treatment wishes and options. Spiritual caregivers also mentioned barriers and facilitators for the provision of spiritual care, such as communication with other healthcare providers, having a relationship of trust and structural funding.. In the online focus group, local multidisciplinary meetings were suggested as ideal opportunities to familiarize other healthcare providers with spirituality and promote spiritual caregivers’ services. Also, structural funding for spiritual caregivers in the primary care setting should be organized.

**Conclusion:**

Spiritual caregivers provide broad spiritual care at the end of life, and discuss many different topics beside spiritual issues with patients in the palliative phase, supporting them when making medical end-of-life decisions. Spiritual care in the primary care setting may be improved by better cooperation between spiritual caregiver and other healthcare providers, through improved education in spiritual care and better promotion of spiritual caregivers’ services.

## Background

Spiritual care, an intrinsic aspect of palliative care [1], is a broad concept for which many definitions exist [2–5]. In 2011, the EAPC taskforce on spirituality adjusted a preceding North American consensus definition [6] and defined it as ‘the dynamic dimension of human life that relates to the way persons (individual and community) experience, express and/or seek meaning, purpose and transcendence, and the way they connect to the moment, to self, to others, to nature, to the significant and/or the sacred.’ [7] The Netherlands Comprehensive Cancer Organisation adopted this definition in their recently revised guideline on spiritual care in palliative care [8]. While religious spirituality, in which religion provides identity, morals and faith in a higher power, may be distinguished from secular spirituality, which emphasises on unity, integrity, holism and individuality [9], this definition comprises both.

Receiving spiritual care is associated with better quality of life for patients with a life-threatening illness [4, 10–12]. As in other countries, a pastor or chaplain used to be the designated spiritual care provider in primary care in the Netherlands, but due to progressing secularisation in the population [13, 14], only a minority of patients is member of a religious society. Currently, in the Dutch coordinated care model of providing palliative care [15], it initially falls to general practitioners and district nurses to assess and address spiritual needs in their patients.

In recent years, a number of international studies have identified a variety of unmet spiritual needs in patients with a life-threatening illness [16–20]. Even though patients are willing to talk about end-of-life issues and spirituality with their healthcare providers [21–23], physicians and nurses struggle to provide it [24–27]. Institutional factors such as high workload and low staffing, personal factors such as lacking attention for spirituality or perceiving a patient’s spirituality as a personal matter, and cultural factors such as religious discordance impede provision of spiritual care [12, 27–30]. Lack of training and care providers not perceiving it as their task have also been shown to be negatively associated with spiritual care provision [31]. When a lack of knowledge, skills or time, or the mere complexity of spiritual issues hampers healthcare professionals in providing adequate spiritual care, they have the option of referring patients to professional spiritual caregivers. In the Dutch primary care setting however, such referral is rare. A study on the involvement of supportive care professionals in the Netherlands found that, in this setting, spiritual caregivers were involved in the care of less than 13% of the patients in the last month of life [32].

Although research explaining the low number of consistent referrals of patients with spiritual issues to spiritual caregivers in the Netherlands is scarce, a recent study showed that physicians and nurses refrain from referring to spiritual caregivers because they do not perceive it as their task or see no added value in their involvement [33]. Research from other countries showed that the unfamiliarity of healthcare providers with spiritual care and spirituality can be an important barrier to referral [26, 34]. For many healthcare providers what the spiritual dimension entails is unclear, as is the role of a spiritual caregiver and how to find one. Improving the understanding of the role of the spiritual caregiver in the primary care setting can lead to more referrals, and ultimately, to better palliative care [10–12]. So, rather than trying to formulate a clear and concise definition of spiritual care, we believe that describing in concrete terms what care provided by spiritual caregivers looks like, may help healthcare providers understand the role of such a caregiver. The primary aim of this study was therefore to investigate and describe the practice of spiritual caregivers in the primary care setting. The secondary aim was to investigate, from their own perspective, the reasons why spiritual caregivers are infrequently involved in palliative care and what they think is needed to improve this.

## Methods

### Design

This study is part of a larger project aimed at improving palliative care in the primary care setting with a sequential mixed methods design. A two-step needs assessment among healthcare providers in the primary care setting was performed. This study focusses on the responding spiritual caregivers within the project.

The first part of the needs assessment was an online questionnaire that was available online from the 5th of April 2016 until the 5th of August 2016, containing open and structured questions on the participants’ most recent case of palliative care. The second part consisted of an online focus group in which the insights from the online questionnaire were explored more in-depth. The online focus group was held on a website with an interface similar to an online chat room. Participants logged into the website, using a code name and password provided by a moderator (IK), where they responded to the questions posed by the moderator and other respondents’ reactions. The website was accessible 24 h a day, from the moment the first question was presented until a week after the last question was presented. When participants clicked on a question, they could read it with its context, read any earlier comments from other respondents and write a response. Any personal information, or information that identified specific individuals or organisations was depersonalised by the moderator.

### Participants

Participants in the online questionnaire were recruited through two professional associations for spiritual caregivers: the Spiritual Caregivers Association (VGVZ), and Humanistic Covenant (HV). Inclusion criteria were: 1) working as a spiritual caregiver in the Netherlands, 2) working in the primary care setting, and 3) having experience with providing palliative care. The professional associations sent the call to participate to 112 and 110 of their members respectively, whom they knew were involved in palliative care in the primary care setting. But as it is likely that these numbers overlap, the actual number of eligible spiritual caregivers that received the call is uncertain. There is also no data available on the number of spiritual caregivers practicing in primary care settings in the Netherlands. In total, 31 spiritual caregivers described the most recent case in which they had provided palliative care. In the final question of the online questionnaire, participants were asked to leave their contact details if they were interested in participating in a focus group aimed to further investigate points of improvement in palliative care. Participants who did, were invited to participate through an e-mail containing information on the procedure, subjects of discussion and the ground rules. In total, 26 were invited, and 16 responded of whom 7 declined. Finally, 9 spiritual caregivers participated in the online focus group.

### Data collection

After some structured questions on the characteristics of their most recent case of palliative care, participants in the questionnaire were prompted to describe their case through three open questions: *1) Can you describe the situation and the palliative care you provided, 2) Can you describe what went well in this case,* and *3) Were there things that could have gone better*. They were also asked to report on eight end-of-life topics whether they had discussed these and if so, with whom. These topics were ‘life expectancy’, ‘complications’, ‘treatment options’, ‘hospital admissions’, ‘palliative sedation’, ‘preferred place of death’, ‘spiritual issues’ and ‘euthanasia’.

Following the questionnaire, eight questions were posed over the course of two weeks in the online focus group. In this paper we focus on two: *1) What causes spiritual caregivers to be infrequently or untimely involved in palliative care*, and *2) In what way can spiritual caregivers contribute to more frequent and earlier involvement.*

### Data analysis

The data from the online questionnaire was analysed through open coding [35]: codes were derived from the data rather than determined beforehand. First, the data was analysed and coded by IK. Second, the codes were discussed with HP, and finally, with all other authors. During this process, codes underwent content and definition changes as the analysis progressed and relations between codes became apparent. From the data, codes in three categories were identified [1]: aspects of care (i.e. the practice of spiritual caregivers) [2], dimensions that are covered (i.e. the dimensions in which care is provided), and [3] barriers and facilitators for the provision of spiritual care.

## Results

### Characteristics of spiritual caregivers

Table 1 provides an overview of the characteristics of the participants, including the subgroup of the online focus group. The majority was female and was working part-time. The participants had a broad range of age and years in practice. While every participant worked in at least one primary care setting (home, care home or hospice), half (16/31) worked in more than one, including hospitals and other settings such as psychiatric wards, institutions for the mentally impaired and rehabilitation centres.

**Table 1:** Characteristics of spiritual caregivers participating in the online questionnaire and online focus group

	Online questionnaire (*N* = 31)	Online focus group (*N* = 9)
Age (years), mean (range)	54 (27–74)	50 (35–63)
Gender	22 F 9 M	8 F 1 M
Working part-time (mean hours)	25 (22)	7 (23)
Years in practice, mean (range)	13 (1–30)	12 (1–30)
Setting^1^		
Home	13	4
Hospice	10	3
Residential home	8	2
Secondary care setting	17	6
Elsewhere	8	0
Denomination^2^		
Christian	19	7
Humanistic	9	3
No institutional affiliation	8	3
Other	1	0
Education in palliative care	15	5
Number of patients cared for in last year, mean (range)	27 (0–120)	34 (2–100)

^1^ Participants could work in more than one setting; other settings included: psychiatric ward, rehabilitation ward, care hotel, monastery, institute for the mentally-impaired

^2^ Participants could have more than one denomination; Christian denominations included: catholic, protestant, oecumenical; other denomination: Buddhist

More than half (19/31) of the participants had a Christian denomination, while nine had a humanistic and one a Buddhist denomination. Eight had no institutional affiliation. Almost half of the participants indicated they had received education in palliative care. The average number of patients at the end of life they cared for in the last year was 27, with a considerable range (0–120).

### Online questionnaire: results from the case descriptions

#### Patient characteristics

Thirty-one spiritual caregivers described their most recent case in which they had provided spiritual care. Table 2 provides an overview of the patients’ and care characteristics of the described cases. The mean age of the described patients was 72 years, and half were female. Most patients were diagnosed with cancer (*n* = 24), eight suffered from organ failure and seven from frailty or dementia. Fifteen patients remained at home while a minority remained in a hospice (*n* = 5) or a residential home (*n* = 3). As some participating spiritual caregivers also worked in other settings (Table 1), and they were asked to describe their most recent case, some of the cases concerned patients in secondary care (*n* = 4), or another setting (n = 2).

**Table 2:** Patient characteristics, N = 31

Age, mean (range)	72 (29–91)
Gender	15 F 15 M
Diagnosis^1^	
Cancer	24
Organ failure	8
Frailty/dementia	7
Unknown	1
Setting^2^	
Home	15
Hospice	5
Residential home	3
Secondary care setting	4
Elsewhere^3^	2

^1^ Patients could have more than 1 diagnosis

^2^ Missing data for 2 patients

^3^ Care hotel or institute for the mentally impaired

From the case descriptions, we derived aspects and dimensions of spiritual care as well as barriers and facilitators for the provision of spiritual care in the primary care setting. Table 3 provides three exemplifying case descriptions in full. An overview of aspects and dimensions of spiritual care with exemplifying quotes can be found in Table 4.

**Table 3:** Exemplifying case descriptions by spiritual caregivers. Cases are anonymized

*R15* *Case*: ‘Mr. A. needed reflection in the form of conversations about his life. Since his incurable illness many questions arose about the purpose and meaning of life and how he could be significant in this stage of life.’ *What went well*: ‘A good connection led to strong relationship of trust. Because of this, Mr. A. could openly talk about his life, his questions and his doubts.’ *What could have gone better*: ‘There was no communication between me and the general practitioner and I missed that in being able to adjust to each other.’	
*R11* *Case*: ‘Mr. B. was bedridden, and had a lot of visitors. I listened to his stories a lot, which went further than daily worries and occurrences.’ *What went well:* ‘He enjoyed talking about more serious topics now and then. He slowly came to some sort of acceptance of what was happening to him.’ *What could have gone better:* ‘Sometimes he wanted to speak freely, and sometimes just a short visit. I could have realized that last part a bit better so I wouldn’t have stayed too long and have the nurse telling me I should visit less often.’	
*R09* *Case*: ‘Mr. C. had attacks of severe pain, itch and dyspnoea. He didn’t want this anymore and talked with the general practitioner about euthanasia, but couldn’t make a decision, because of an inner conflict with his religious values. In conversation with Mr. C., his partner and daughter, I clarified the situation, values and (religious) coping style of Mr. C., after which he could come to an informed decision.’ *What went well:* ‘The opinions and values of Mr. C. were openly discussed, without any pressure into a certain direction. As well as the concern for his wife and daughter, and the burden Mr. C. thought to be, as a possible factor in the decision-making process. Also, good communication with the general practitioner.’ *What could have gone better:* ‘Prior information on an alternative, palliative sedation, could have been more clear.’

**Table 4:** Aspects and dimensions of care provided by spiritual caregivers

Category 1: Aspects of spiritual care and exemplifying quotes	
Helping to find meaning, acceptance or reconciliation	
- *She accepted the fact she was going to need increasingly more help and that she eventually was going to die. (R10)*	
- *He felt heard and had the specific question for me to help him learn to pray again. Additionally, I helped him realize he wanted to ask forgiveness from his wife and son for his alcohol abuse in the past. (R26)*	
Attention for patient’s relative(s)	
- *In separate conversations with the patient and his spouse, it turned out that the patient’s demise was a difficult subject. I facilitated a conversation between patient and wife about the coming death. (R14)*	
- *The children disagreed about the treatment plan. We talked about their thoughts, expectations and fears and the underlying pain and grief from the death of their other parent 16 years ago. Taking time for their suffering. (R25)*	
Performing a (farewell-)rite	
- *Good guidance, a farewell-ritual with children. Let go of life and died three days later. (R04)* - *Only one granddaughter was present at the time of the farewell rite. Based on the son’s description of his mother, I read a poem about saying farewell, a prayer of Mary and I asked the granddaughter to tell her grandmother what she was grateful for. Finally, we prayed. Madam seemed unconscious for the greater part of the rite, but at the end of the prayer she said a heartfelt ‘amen’. (R17)*	
Helping to say farewell	
- *She opened up, was able to enjoy things and she said goodbye to her family and friends very consciously. In the end there was surrender and faith that everything was alright. (R08)* - *I began visiting him weekly where we spoke about the end of life, saying farewell, and ways to inform loved ones* et cetera*. (R20)*	
Acknowledgement (n = 5)	
- *He felt recognized and heard because of the respectful and reticent position I took regarding my own way of thinking. (R27)* - *Being around him, connecting to his world, talking about the place after the end of this life. Offering encouragement and trust. Comradery, ganging up together. (R01)*	
(Help) organizing the funeral	
- *I discussed preparations for the funeral with him and organized it along with the children. (R21)* - *I met the family and talked to them. It was nice to be able to do the funeral in cooperation with the companies, wife and family. (R05)*	
Spiritual counselling (not specified)	
- *She and her son wanted counselling on a spiritual level and I was able to provide this. (R07)* - *I guided him spiritually and I had conversations with him about his life and its conclusion, and his wishes and expectations. (R16)*	
Category 2: Dimensions of care and exemplifying quotes	
Existential	
- *I came to talk with them about the illness of the husband, what it meant for him, what he still wanted in life. And also for the wife: how to spend time together, how to say goodbye* etcetera*. (R12)* - *I guided the patient in looking back on her life, existential questions and in the terminal phase by being there and conversing and supporting the caregiver and her family. (R24)*	
Relational	
- *The patient was very concerned with the future of her partner, at first this eclipsed her own process of dying. I had weekly conversations with her, and later also with her daughter and granddaughter. (R19)* - *She wanted to be eligible for euthanasia in order to not be a burden for her children, and because of her fear of pain and death. When her wish was declined, I assisted her in accepting that. There were also some feelings of anger and resentment towards her son-in-law, the husband of her deceased daughter. I assisted her in managing this. (R31)*	
Religious	
- *Madam used to be member of a church denomination that ended in the previous century and she found out her belief system didn’t work anymore. This increased her anxiety. As spiritual caregiver I provided her a listening ear and understanding. I could also assist her in her way of seeking religious answers. These conversations gave her consolation. (R31)* - *He became a Buddhist in the final five years of his life. I guided him in his existential questions which he approached either from the more traditional Christian framework from his ‘former’ life or from his recent search for Buddhist answers. (R27)*	

#### Aspects of spiritual care

In most case descriptions, we found more than one aspect of spiritual care in practice. In total, we distinguished six separate aspects of spiritual care from the case descriptions. These aspects were [1]: helping the patient to find meaning, acceptance or reconciliation [2], having attention for relatives of the patient, and [3] performing a (farewell) rite to be part of the spiritual care they provided [4]. Helping to say farewell [5], acknowledgement, and [6] organizing the funeral were also described as part of provided care. Also, some spiritual caregivers stated they had provided spiritual care, but did not go into detail.

#### Dimensions of spiritual care

From the data we distinguished three dimensions covered by spiritual care. Similar to the aspects, we found that in most cases the provided care covered more than one, due to the multidimensional issues of the patient. The descriptions that spiritual caregivers gave of their care were distinguished by us in tending to their patient’s [1] existential issues, concerning for example hope, suffering or the meaning of life and illness to oneself [2], relational issues, concerning (still) being of value to or the significance of relatives and [3] religious issues in their patient, concerning one’s beliefs and practices or (a loss of) faith in God or a higher power. In addition, spiritual caregivers described tending to the emotions patients experience related to the existential, relational or religious issues they face.

#### End-of-life topics discussed

**Fig. 1:**
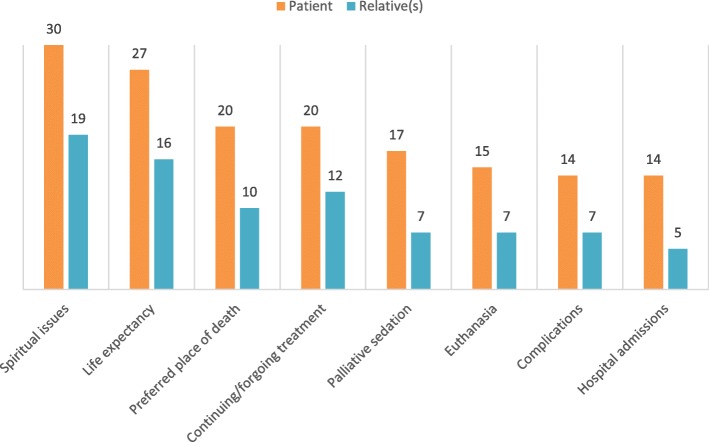
Topics discussed by spiritual caregivers with patients (*n* = 30) and relatives (*n* = 26)

We also asked the participants to indicate whether they discussed eight specific end-of-life topics with the patients and/or their relatives. Figure 1 provides an overview of these topics. While spiritual issues and life expectancy are discussed with almost all patients and the majority of relatives, all other topics are also addressed regularly. Spiritual caregivers discussed topics related to medical care and treatment, such as treatment options, complications and hospital admissions with their patients and to a lesser extent with their relatives.

#### Barriers and facilitators for the provision of good spiritual care

Besides the aspects and dimensions of care, we also derived other factors relevant to spiritual care provision from the data. These issues, sometimes mentioned as something that went well, sometimes as something that could have gone better, facilitated or hampered spiritual caregivers in the provision of care, but were not described as part of the provided care.

Facilitators that were mentioned included communication with other healthcare providers, a relationship of trust with the patient and communication with the patient, while the absence of structural funding, the lack of knowledge of spiritual care in other healthcare providers, being involved too late were referred to as barriers. Another factor the participants indicated to play a role in the provision of spiritual care was dosage of care (adjusting the amount of time spent with the patient to his or her wishes).

### Online focus group: reasons spiritual caregivers are not involved and how to improve involvement

In the online focus group, we asked the participants why spiritual caregivers in general are infrequently involved in palliative care in the primary care setting, and what they can do to be involved more often, and in time. Table 5 provides an overview of the reported reasons and suggestions for improvement.

**Table 5:** Reasons spiritual caregivers are infrequently involved in primary care and suggestions for improvement

Reasons spiritual caregivers are infrequently involved	
1. Other healthcare providers have insufficient knowledge of spiritual care	
2. Other healthcare providers do not know spiritual caregivers or how to find one	
3. Spiritual care is not funded in primary care	
Suggestions to improve involvement of spiritual caregivers	
1. Training of healthcare providers in primary care in recognizing spiritual distress	
2. Active promotion of spiritual caregiver services in primary care to increase awareness of their availability	
3. Organise structural funding / insurance coverage in primary care	

Participants in the online focus group indicated that, according to them, other healthcare providers have insufficient knowledge of and attention for spiritual care and do not know spiritual caregivers in person or how to find one. They suggested a twofold solution: healthcare providers should be better trained in recognizing spiritual distress and when to refer, while spiritual caregivers should promote their services better and making their availability more widely known. Participating in multidisciplinary meetings was suggested as a way to do so. The lack of funding for spiritual caregivers in primary care was also raised as an issue, with an apparent solution: organising insurance coverage.

## Discussion

### Reflections on the aspects and dimensions of spiritual care

This is, to our knowledge, the first empirical study describing the practice of spiritual caregivers for patients at the end of life in the primary care setting from their perspective. Spiritual caregivers provide care in several dimensions, including care for existential, relational and religious issues. We identified a wide variety aspects of this care in practice, including helping patients find meaning, acceptance or reconciliation, paying attention to the spiritual issues of relatives of the patient, and helping them all to say farewell. Other aspects we identified were performing rites, helping with the funeral or just simply ‘being there’.

In a recent study on the practice of spiritual caregivers working in palliative care in hospitals in the US, similar aspects of spiritual care were found [36]. Spiritual caregivers in that study provided spiritual care in many ways, including providing ritual support, caring for relatives, facilitating communication between patient/families and care teams and addressing familial conflicts. Still there was one notable difference in that the US chaplains emphasized religious distress more than the participants in our study. This may be explained by the fact that religion plays a larger role in the US than it does in the Netherlands [14, 37].

Regarding the dimensions of spiritual care, earlier research by The EAPC Task Force on spiritual care stated that spirituality comprises three dimensions: (i) existential questions, (ii) religious considerations, and (iii) value-based considerations (i.e. (inter) relational issues) [7]. Although the European Organisation for Research and Treatment of Cancer (EORTC) Quality of life group phrased the latter differently, it mentioned the same three dimensions of spiritual well-being [38]. In our data, we found that spiritual caregivers also deal with their patients’ emotional issues. But rather than proposing emotional issues as a fourth dimension, we feel that these emotions like anger, anxiety, and grief are intertwined with the three dimensions and cannot be seen as separate.

### Reflections on topics of discussion

The participating spiritual caregivers indicated for eight topics whether they discussed these, showing the wide range of topics that spiritual caregivers talk about with patients and their relatives. Similar to the aspects of spiritual care in practice, the topics discussed are diverse. Naturally, spiritual issues are discussed, but other topics like life expectancy, treatment options and preferred place of death are also discussed regularly. As other healthcare providers are also likely to talk about these topics with patients at the end of their lives, it would be interesting to study the added value of discussing these from a different vantage point.

### Reflections on barriers and facilitators

Spiritual caregivers also mentioned barriers and facilitators for the provision of spiritual care, such as communication with other healthcare providers, having a relationship of trust and communication with the patient. Some of these factors are mentioned as conditions for the provision of spiritual care in the Dutch guideline on spiritual care in palliative care [8]. The guideline recommends healthcare providers to take time for the spiritual issues of their patient, be open, build a relationship of trust and compassion and respect their own limitations. Similarly, in the previously mentioned study on the practice of chaplains in the US, building relationships was found to be the primary activity [36].

Although the guideline on spiritual care provision recommends that healthcare providers refer to specialist spiritual caregivers in case of suspected existential or spiritual crises [8], earlier research in the Netherlands which showed that general practitioners and spiritual caregivers rarely cooperate in palliative care [39]. Communication between healthcare providers as mentioned by the spiritual caregivers in our study, unfortunately remains unaddressed in the guideline, even though it could be critical in the shift from multidisciplinary to interdisciplinary care.

### Reflections on suggestions to improve referral

The spiritual caregivers in the online focus group mentioned that more familiarity of other healthcare providers with spirituality and spiritual caregivers is likely to lead to more referrals. Local multidisciplinary meetings were suggested as ideal opportunities to familiarize other healthcare providers with spirituality and promote spiritual caregivers’ services. Interestingly, in the guideline on spiritual care, healthcare providers are encouraged to invite a spiritual caregiver to permanently join their multidisciplinary meetings to ensure attention for the spiritual dimension of palliative care [8]. The recent establishment of regular funding the structural engagement of spiritual caregivers in primary care, both directly with patients and indirectly through multidisciplinary meetings [40], will arguably make this more feasible. Future research should examine whether this development indeed leads to an improvement in spiritual care provision to patients.

The participating spiritual caregivers also mentioned that training other healthcare providers in recognizing spiritual care needs can lead to adequate referral. This is in line with results from a recent study, which showed a positive effect of spiritual care training on the attitudes and competencies of Dutch hospital staff, including an increase of referrals to spiritual care specialists [41]. Still, this effect may be smaller in the primary care setting, where healthcare providers do not work in the same institution and thus may have more difficulty finding each other.

### Strengths and limitations

A strength of this study is that we asked the field to describe their practice and we used their descriptions to derive a variety of important aspects of and conditions for spiritual care. A limitation of this study is that although we feel that the study provides a relevant overview of spiritual care in the primary care setting, it is uncertain whether we reached data saturation. Firstly, as we derived the case descriptions from a questionnaire, we could not ask probing questions regarding a specific case. Secondly, we were only able to include spiritual caregivers with a North-western European understanding of spirituality. As the Netherlands is a multicultural society, harbouring people with various understanding of spirituality, this description of spiritual care in the Netherlands may be incomplete. For instance, as the Islamic view on life and death differs greatly from the Western biomedical view [42], spiritual care for Islamic patients may have a different approach, with different aspects playing a role and different topics being discussed at the end of life [43, 44]. We recommend extra attention to minority groups in future research on spiritual care in the Netherlands.

### Practical implications

Although patients have unmet spiritual needs at the end of life, spiritual caregivers are infrequently involved in the primary care setting, due to unfamiliarity of healthcare providers with spirituality and the role of spiritual caregivers in palliative care. The overview of the practice of spiritual caregivers in this paper may provide healthcare providers such as general practitioners and district nurses with insight in the practice of spiritual caregivers. This may lead to better understanding of the added value of a spiritual caregiver in a particular patient, possibly leading to more appropriate palliative care for patients with spiritual care needs. In addition, this paper identifies opportunities to increase (timely) referrals in patients with spiritual distress. According to the field, better training of other healthcare providers in recognizing spiritual needs and a more proactive approach in the promotion of their services is needed, and spiritual caregivers joining local multidisciplinary meetings may be a good way to achieve this. The information derived from this study is used in a current study on the feasibility of involving spiritual caregivers in PaTz-groups [45], local multidisciplinary groups in the Netherlands aimed at improving palliative care in the primary care setting.

Finally, while the role of spiritual caregivers in this setting became more important as secularisation progressed, their funding has, until lately, been overlooked. Time will tell whether recent funding developments [40] will improve the involvement of spiritual caregivers, and the provision of spiritual care for patients in the primary care setting.

## Conclusion

Spiritual caregivers provide broad spiritual care at the end of life and may have added value in palliative care. In order to provide adequate spiritual care in palliative care, better cooperation between spiritual caregivers and other healthcare providers in the primary care setting is needed. This requires further promotion of spiritual caregivers’ services and more education in spiritual care for other healthcare providers. Recent funding developments to improve the engagement of spiritual caregivers in the primary care setting may be supportive in this respect.

## Data Availability

The dataset used and analyzed during the current study are available from the corresponding author on reasonable request.

## Abbreviations

EAPC: European Association for Palliative Care
EORTC: European Organisation for Research and Treatment of Cancer
HV: Humanistisch Verbond (Humanistic Convenant)
PaTz: Palliatieve ThuisZorg (Palliative Care at Home)
VGVZ: Vereniging van Geestelijk Verzorgers (Spiritual Caregivers Association)
